# Targeting antisense mitochondrial ncRNAs inhibits murine melanoma tumor growth and metastasis through reduction in survival and invasion factors

**DOI:** 10.18632/oncotarget.11110

**Published:** 2016-08-06

**Authors:** Lorena Lobos-González, Verónica Silva, Mariela Araya, Franko Restovic, Javiera Echenique, Luciana Oliveira-Cruz, Christopher Fitzpatrick, Macarena Briones, Jaime Villegas, Claudio Villota, Soledad Vidaurre, Vincenzo Borgna, Miguel Socias, Sebastián Valenzuela, Constanza Lopez, Teresa Socias, Manuel Varas, Jorge Díaz, Luis O. Burzio, Verónica A. Burzio

**Affiliations:** ^1^ Andes Biotechnologies SpA, Santiago, Chile; ^2^ Fundación Ciencia & Vida, Santiago, Chile; ^3^ Facultad de Medicina, Universidad de Chile, Independencia, Santiago, Chile; ^4^ Facultad de Ciencias Biológicas, Universidad Andrés Bello, República, Santiago, Chile; ^5^ Facultad de Salud, Deporte y Recreación, Universidad Bernardo O'Higgins, Santiago, Chile; ^6^ Servicio de Urología, Hospital Barros-Lucco-Trudeau, Santiago, Chile; ^7^ Clínica Alemana, Santiago, Chile; ^8^ Present address: Facultad de Ciencias Biológicas, Pontificia Universidad Católica de Chile, Santiago, Chile

**Keywords:** mitochondria, noncoding RNA, melanoma, metastasis, antisense therapy

## Abstract

We reported that knockdown of the antisense noncoding mitochondrial RNAs (ASncmtRNAs) induces apoptotic death of several human tumor cell lines, but not normal cells, suggesting this approach for selective therapy against different types of cancer. In order to translate these results to a preclinical scenario, we characterized the murine noncoding mitochondrial RNAs (ncmtRNAs) and performed *in vivo* knockdown in syngeneic murine melanoma models. Mouse ncmtRNAs display structures similar to the human counterparts, including long double-stranded regions arising from the presence of inverted repeats. Knockdown of ASncmtRNAs with specific antisense oligonucleotides (ASO) reduces murine melanoma B16F10 cell proliferation and induces apoptosis *in vitro* through downregulation of pro-survival and metastasis markers, particularly survivin. For *in vivo* studies, subcutaneous B16F10 melanoma tumors in C57BL/6 mice were treated systemically with specific and control antisense oligonucleotides (ASO). For metastasis studies, tumors were resected, followed by systemic administration of ASOs and the presence of metastatic nodules in lungs and liver was assessed. Treatment with specific ASO inhibited tumor growth and metastasis after primary tumor resection. In a metastasis-only assay, mice inoculated intravenously with cells and treated with the same ASO displayed reduced number and size of melanoma nodules in the lungs, compared to controls. Our results suggest that ASncmtRNAs could be potent targets for melanoma therapy. To our knowledge, the ASncmtRNAs are the first potential non-nuclear targets for melanoma therapy.

## INTRODUCTION

Human cells express a unique family of sense and antisense non-coding mitochondrial RNAs (ncmtRNAs) [1–3]. The sense transcript (SncmtRNA) is expressed in normal proliferating cells and tumor cells, but not in resting cells, suggesting a role of this transcript in cell proliferation [1–3]. Besides SncmtRNA, normal proliferating cells express two antisense transcripts: ASncmtRNA-1 and ASncmtRNA-2 [2]. Remarkably, the ASncmtRNAs are downregulated in human tumor cell lines and tumor cells present in biopsies of different types of cancer [2]. Hence, downregulation of the ASncmtRNAs seems to be an important step in carcinogenesis and represents a new and generalized pro-tumorogenic hallmark of cancer [4]. Other mitochondrial transcripts also containing stem-loop structures have been reported, although their function is unknown [5].

Recently, we showed that knockdown of ASncmtRNAs (ASK for short), using antisense oligonucleotides (ASO), induces apoptotic cell death of several human cancer cell lines, including SK-MEL-2 melanoma cells [6]. How do we explain that ASK is efficient in inducing knockdown of the ASncmtRNAs since it is well known that oligonucleotides are not able to enter mitochondria *in vivo* [7, 8]? Previously, we have demonstrated that in normal human kidney, renal cell carcinoma, mouse testis and the murine melanoma cell line B16F10, the SncmtRNA and the ASncmtRNAs exit the mitochondria and are found localized in the cytoplasm and in the nucleus [9]. We used several approaches, including electron microscopy *in situ* hybridization (ISH), and these results suggest that the functional role of these molecules lies outside the organelle [9]. Perhaps the nuclear localization suggests that these transcripts might be new players in the mitochondrial-nuclear communication pathway or retrograde signaling [10].

ASK also induces downregulation of the cytoprotective factors survivin and XIAP [6], members of the inhibitor of apoptosis protein (IAP) family, which are upregulated in virtually all human cancers, including melanoma [11–15]. Considering our results on human melanoma *in vitro* [6], the real challenge was whether translation of these results to an *in vivo* preclinical scenario with immunocompetent mice would inhibit melanoma tumor growth. Besides the characteristics of the murine ncmtRNAs (mSncmtRNA and two mASncmtRNAs), here we show that the mASncmtRNAs are also downregulated in murine melanoma B16F0 and B16F10 cells and murine renal cancer RenCa cells. Similarly, ASK with ASO targeted to the mASncmtRNAs induces B16F10 apoptotic cell death *in vitro,* concomitantly with survivin downregulation. For syngeneic *in vivo* studies, we used B16F10 cells, a highly aggressive and metastatic murine melanoma cell line. We observed a decrease in subcutaneous B16F10 melanoma tumor growth rate in C57BL/6 mice. Furthermore, we used a preclinical approach similar to the clinical practice guidelines of melanoma: surgical resection of the lesion followed by ASK [16–20]. For this purpose, Subcutaneous B16F10 tumors (700 to 1,000 mm^3^) were surgically removed at 11-12 days post-cell injection and mice were then systemically treated with ASO-1560S, complementary to the mASncmtRNAs. Compared to controls, the specific ASO markedly inhibited tumor growth and metastasis to the lung and liver. In a classical metastasis assay, cells were injected through the tail vein and treatments were performed as well by systemic administration of ASOs. ASO-1560S decreased the number and size of metastatic nodules in the lungs. Therefore the ASncmtRNAs may be clinically relevant as targets to treat melanoma.

## RESULTS

### Characteristics of the mouse mitochondrial ncRNAs

**Figure 1:**
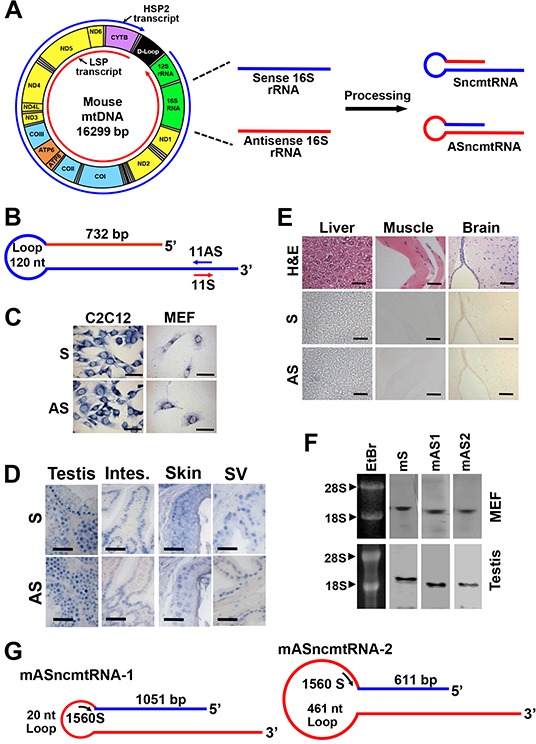
Deduced structure and expression of the mouse ncmtRNAs **A.** Model of the origin of ncmtRNAs. A simplified scheme of mitochondrial transcription shows that both strands of the mtDNA are transcribed bidirectionally. Shown in red is the transcript arising from the light strand (L-strand) promoter (LSP) and in blue, the transcript from the heavy strand (H-strand) promoter HSP2. For simplification, the H-strand transcript arising from HSP1 is not shown. Segments originated from the 16S rRNA gene are then processed to give rise to SncmtRNA and ASncmtRNA. Also shown is the 12S rRNA gene and coding genes for proteins of the NADH dehydrogenase complex (ND1, ND2, ND3, ND4, ND4L, ND5 and ND6), cytochrome oxidase complex (COI, COII and COIII), ATP synthase complex (ATP6 and ATP8) and cytochrome B; the 22 tRNAs are shown in grey. **B.** Schematic representation of the mSncmtRNA, indicating the size of the loop, the length of the IR and position of oligonucleotides 11S and 11AS used as probes. **C.** ISH of C2C12 mouse myoblasts and mouse embryonic fibroblasts (MEF) showing a positive signal for the 11AS probe (B), confirming the expression of the mSncmtRNA, and a positive hybridization signal also with the 11S probe (B), corresponding to a putative mASncmtRNA. Bars = 25 μm. **D.** ISH of sections of normal mouse tissues, testis, intestine (Intes.), skin and seminal vesicles (SV) with probes 11S for mASncmtRNA and 11AS for mSncmtRNA. Hybridization signal was observed with both probes. Bars = 50 μm. **E.** No ISH signal was observed in normal mouse liver, muscle or brain sections, for probe 11AS (S) or 11S (AS). A parallel section was stained with H & E. Bars = 50 μm. **F.** Northern blot of total MEF and testis RNA using probes for mSncmtRNA (S) and mASncmtRNAs (mAS1 and mAS2) (see Materials and Methods). EtBr, ethidium bromide-stained gel. **G.** Schematic representation of mASncmtRNA-1 and −2, indicating the length of the loops and the IRs. ASO-1560S, complementary to both loops, was used for knockdown of these transcripts.

Analogous to the human counterparts, murine ncmtRNAs should arise from the bidirectional transcription from the light and heavy strands of the mitochondrial genome [21]. Processing of the segments from the 16S rRNA gene should give rise to mouse SncmtRNA (mSncmtRNA) and ASncmtRNA (mASncmtRNA) (Figure 1A). These transcripts were characterized by the “PCR-walking” method previously reported for the human ncmtRNAs [1–3] (Supplementary Figure S1A, S1B). The structure of the sense transcript (mSncmtRNA; Genbank Accession AF089815.2) was obtained by RT-PCR of RNA from C2C12 mouse myoblasts, using reverse primer 1 together with forward primers 2-9, yielding a ladder of amplicons (Supplementary Figure S1B). The 766 bp fragment amplified with primer 8 comprises an IR of 732 nt linked to the first 33 nt of the 5′ end of the mouse 16S mitochondrial rRNA (mtrRNA) (details in legend of Supplementary Figure S1). The sequence of the other smaller fragments (Supplementary Figure S1B) revealed that they were part of the same RNA. Since the IR is fully complementary to an internal sequence of the 16S mtrRNA, the transcript should be 2,290 nt in length and contain a 732 bp double-stranded stem and a 120 nt loop (Figure 1B). As expected, the stem was resistant to RNase A digestion but not the single-stranded loop, since post-RNase A treatment, amplification was obtained only with primers complementary to the double-stranded regions of the transcripts [1–3] (Supplementary Figure S1C).

ISH with digoxigenin-labeled primer 11AS, complementary to the mSncmtRNA (Figure 1B) reveals that mouse myoblast C2C12 cells and mouse embryonic fibroblasts (MEF) express this transcript (S, Figure 1C). However, a strong hybridization signal was also observed with the sense probe (primer 11S, Figure 1B) used as control (AS, Figure 1C), suggesting that mouse normal proliferating cells express an antisense counterpart of mSncmtRNA, analogously to what we reported for human cells [1–3]. ISH on normal mouse tissues containing proliferating cells including testis, intestine, skin and seminal vesicles also revealed hybridization signals with both probes (Figure 1D). However, no hybridization signal was observed with either probe in tissues with low proliferation rate such as liver, muscle and brain cortex (Figure 1E).

To confirm the ISH results, we performed Northern blot of MEF and mouse testis RNA under denaturating conditions. Detection of each transcript was performed using biotinylated probes spanning the joining region between the 16S (sense or antisense) sequence and the IR of each RNA (see Materials and Methods). In both MEF and testis RNA, we detected the sense (mS) and the antisense (mAS1 and mAS2) transcripts, in bands that migrated above the 18S rRNA (1.87 kb), suggesting that the probes utilized were not simply hybridizing to the 16S rRNA (1.58 kb) (Figure 1F).

We amplified and sequenced these putative antisense transcripts, by “PCR walking”. First, a theoretical antisense transcript was designed, where the antisense 16S RNA transcribed from the L-strand of the mitochondrial 16S gene is linked at its 5′ end to a putative IR corresponding to a fragment of the 16S mtrRNA (Supplementary Figure S2A) [1–3]. RT-PCR walking with C2C12 RNA using reverse primer 12 and forward primers 15, 16, 18, 19, 20, 21 and 22 (Supplementary Figure S2A) reveals a ladder of fragments corresponding to two antisense transcripts (Supplementary Figure S2B). The transcript containing the 1084 bp fragment obtained with primers 12 and 21 was named mASncmtRNA-1 (Genbank Accession GU332588.1) (Supplementary Figure S2B, *). Sequencing revealed a 1051-nt IR linked to the 5′ end of the antisense 16S RNA, forming a 20-nt loop (Figure 1G). The sequence of the other fragment of 642 bp revealed an IR of 611 nt linked to the 5′ end of the antisense 16S mtrRNA (Supplementary Figure S2B, **), with a 461-nt loop; the corresponding transcript was named mASncmtRNA-2 (Figure 1G) (Genbank Accession GU332589.1). The IRs of these transcripts originate double-stranded stems of 1051 and 611 bp, respectively (Figure 1G). As expected, these double-stranded regions are resistant to digestion with RNase A, but not the single-stranded loops (Supplementary Figure S2C, S2D, S2E).

### Knockdown of mASncmtRNAs induces apoptosis of B16F10 cells

**Figure 2:**
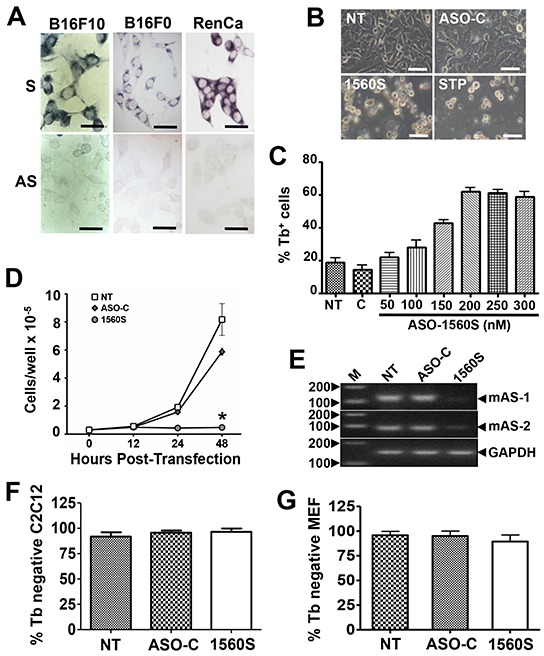
ASK induces death and proliferative index decrease in B16F10 cells **A.** ISH of the murine melanoma cell lines B16F10 and B16F0 and renal carcinoma cell line RenCa, using probes 11S and 11AS (Figure 1A). Notice that, in contrast with normal cells, the mASncmtRNAs are downregulated in the three mouse tumor cell lines. Bars = 25 μm. **B.** B16F10 cells were transfected for 48 h with 200 nM ASO-C, or ASO-1560S, or left untreated (NT). At 48 h post-transfection, ASO-1560S caused massive cell detachment from the substrate, as opposed to controls (NT, ASO-C). STP was included as positive control. Bars = 50 μm. **C.** Cells were transfected with 200 nM ASO-C or with 100, 150, 200, 250 or 300 nM ASO-1560S, or left untreated for 48 h and cell death was determined by Trypan blue (Tb) exclusion. ASK with 200-300 nM ASO-1560S induces over 60% of cell death, compared to 15-20% in NT and ASO-C controls. **D.** B16F10 cells transfected with ASO-1560 or ASO-C as in (B) or left untreated for 48 h were collected at different time intervals. ASO-1560S induces a drastic inhibition of cell proliferation compared to NT and ASO-C controls (NT) (*p<0.01). **E.** RT-PCR of total RNA purified from cells treated as in (B) for 48 h revealed the specific knock-down effect of ASO-1560S on mASncmtRNA-1 (mAS-1) and mASncmtRNA-2 (mAS-2). GAPDH mRNA was used as internal control. M, 100-bp ladder. **F, G.** Normal mouse cells are not affected by ASK. C2C12 (F) and MEF cells (G) were seeded at 5×10^4^ cells/well in 12-well plates and transfected the next day with ASO-C or ASO-1537S or left untreated (NT). After 48 h, cells were harvested and stained with Tb. A triplicate analysis shows that viability of these two non-tumoral mouse cell types was not affected by ASK. Error bars represent average ± s.d.

Analogous to what we reported before for human cancer cell lines [1, 2], ISH of murine tumor cell lines such as B16F0 and B16F10 (melanoma) and RenCa (renal cell carcinoma) results in a positive signal for probe 11AS, but negative for the 11S probe, suggesting that murine tumor cells express the mSncmtRNA but downregulate the antisense version (Figure 2A).

As described before, knockdown of the ASncmtRNAs induces apoptotic cell death in several human cancer cell lines including SK-MEL-2 melanoma cells [6]. Therefore, we determined whether ASK would elicit death in the highly aggressive murine melanoma cell line B16F10. Transfection of B16F10 cells with 200 nM ASO-1560S (phosphorothioate backbone) [6, 22], targeted to the loop of the mASncmtRNAs (Figure 1G), induced marked detachment of cells from the substrate, similar to the effect of staurosporine (STP), while untreated cells (NT) or cells transfected with the control (ASO-C) showed no visible alteration (Figure 2B). ASO-1560S treatment for 48 h induced cell death, as determined by Trypan blue (Tb) exclusion, in a concentration-dependent manner, with a maximum of around 60% using 200 nM ASO. Higher concentrations of ASO-1560S did not enhance cell death (Figure 2C). In comparison, untreated cells or cells transfected with 200 nM control ASO (ASO-C) did not display significant death rates (Figure 2C). Cell death was concomitant with a strong decrease in proliferation of cells transfected with 200 nM ASO-1560S (Figure 2D). RT-PCR confirmed the knockdown of the two mASncmtRNAs (Figure 2E).

Interestingly, viability of non-tumor C2C12 (murine myoblasts) or MEF (mouse embryonic fibroblasts) cells was unaffected by transfection of 200 nM ASO-1560S for 48 h (Figure 2F, 2G). Moreover, C2C12 myoblasts treated with ASO-1560S showed no signs of altered morphology or proliferation, even after 3 days of transfection, as untreated and ASO-C controls (Supplementary Figure S3A). When these cells were washed and induced to differentiate (see Materials and Methods), ASO-1560S-treated cells formed myotubules with normal morphology (Supplementary Figure S3B), which retained normal contractile function (Supplementary Video S1), as observed in cells transfected with ASO-C (Supplementary Video S2) or untreated cells (Supplementary Video S3).

**Figure 3:**
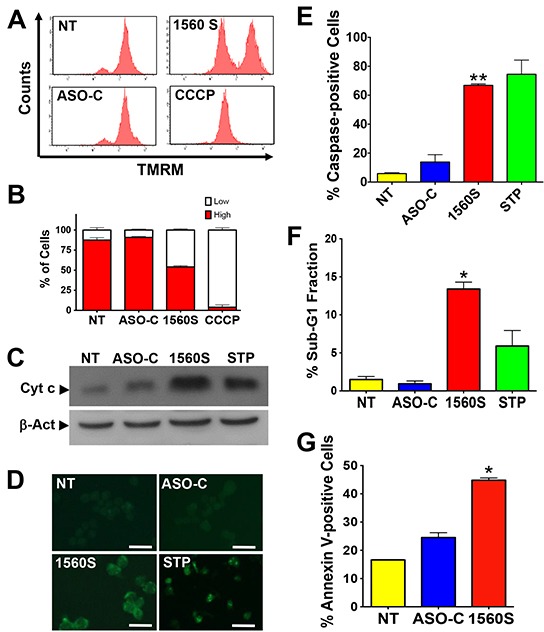
Death of B16F10 cells by ASK displays parameters of apoptosis **A.** ASK induces dissipation of Δψm. B16F10 cells were transfected with 200 nM ASO-C or ASO-1560S, or left untreated for 24h. A parallel culture was incubated with the uncoupling drug CCCP (see Materials and Methods). Cells were then harvested at 24 h post-transfection, stained with 20 nM TMRM for 15 min and analyzed by flow cytometry. ASK and CCCP induce dissipation of Δψm. **B.** A triplicate analysis shows that ASO-1560S induces about 55% dissipation of Δψm compared to 95% for CCCP. **C.** ASK induces release of cytochrome c. B16F10 cells treated as described in (A) or incubated with 5 μm STP as positive control were harvested at 24 h post-transfection. Western blot of the cytosolic fraction (see Materials and Methods) reveals, as compared to controls, a significant release of cytochrome c to the cytosol in cells transfected with ASO-1560S or incubated with STP. **D.** ASK induces caspase activation. B16F10 cells treated as in (A) for 24h were labeled with FITC-VAD-fmk (see Materials and Methods). Parallel cultures were incubated with 5 μM STP as positive control for apoptosis. Only cells treated with ASO-1560S or STP exhibited green fluorescence due to caspase activation. Bars = 25 μm. **E.** A triplicate analysis shows that ASK and STP induce over 60% of cells with activated caspases as compared to controls (**p<0.01). Error bars represent average ± s.d. **F.** ASK induces an increase of sub-G1 fraction. B16F10 cells were treated as in (A), but for 48 h. A parallel culture was incubated with STP as positive control. Cells were then harvested, stained with PI and analyzed by flow cytometry. Only ASO-1560S or STP elicited a significant increase in sub-G1 DNA fraction of 13% and 6%, respectively (*p<0.005). **G.** ASK induces annexin V-positive cells. B16F10 cells were treated for 24 h as in (A), labeled with annexin V-Alexa Fluor 488 and analyzed by flow cytometry. A triplicate analysis shows that ASO-1560S induced about 45% annexin-positive cells as compared to controls (NT and ASO-C) (*p<0.005).

Next, we explored whether cell death elicited by ASK occurs through an apoptotic pathway, as is the case for human cells [6]. Dissipation of the mitochondrial membrane potential (Δψm) is an early step in apoptosis [6, 23–25]. To determine the effects of ASK on Δψm, B16F10 cells were transfected with 200 nM ASO-1560S or ASO-C or left untreated for 48 h. Cells were harvested and incubated with the fluorescent probe tetramethylrhodamine methyl ester (TMRM). The uncoupling agent carbonyl cyanide *m*-chlorophenyl hydrazone (CCCP) was used as a positive control [23]. Flow cytometry revealed that ASO-1560S treatment caused a marked dissipation of Δψm, as with CCCP, while no major change was observed with 200 nM ASO-C or in untreated cells (Figure 3A). Quantitative analysis of three independent experiments shows that ASO-1560S and CCCP induce about 55% and 95% dissipation of Δψm, respectively, compared to 10-15% in controls (Figure 3B). Dissipation of Δψm correlates with cytochrome c release from mitochondria [6, 23–25]. B16F10 cells were transfected for 48 h as described above, including cells incubated with STP as positive apoptosis control. Cells were harvested and processed to obtain the cytosolic fraction [6]. Western blot analysis revealed a considerable increase of cytosolic cytochrome c in cells treated with STP or 200 nM ASO-1560S, compared to controls (Figure 3C). To determine whether ASK triggers caspase activation, B16F10 cells were transfected for 24 h as described or treated with STP. Cells were then incubated with the fluorescent pan-caspase inhibitor FITC-VAD-fmk, which binds to activated caspases [26]. Fluorescent cells were found only after transfection with 200 nM ASO-1560S or incubated with STP (Figure 3D). A triplicate analysis of cells transfected for 24 h revealed that over 60% of cells treated with ASO-1560S or STP contained activated caspases, compared to 5-15% in controls (Figure 3E).

ASK also induces other hallmark apoptotic events [27]. A 48 h treatment of B16F10 cells with 200 nM ASO-1560S induced DNA fragmentation as evaluated by flow cytometric determination of the sub-G1 fraction. A triplicate study indicated that ASK induces about 13% sub-G1 population, while STP induces about 6% and controls were around 1-2% (Figure 3F). In addition, ASK for 48 h induced translocation of phosphatidylserine to the outer layer of the plasma membrane. B16F10 cells treated in triplicate were harvested, incubated with Alexa fluor 488-conjugated Annexin V, counter-stained with PI and analyzed by flow cytometry. ASK induced about 45% Annexin V-positive cells compared with controls (Figure 3G).

### Survivin downregulation

**Figure 4:**
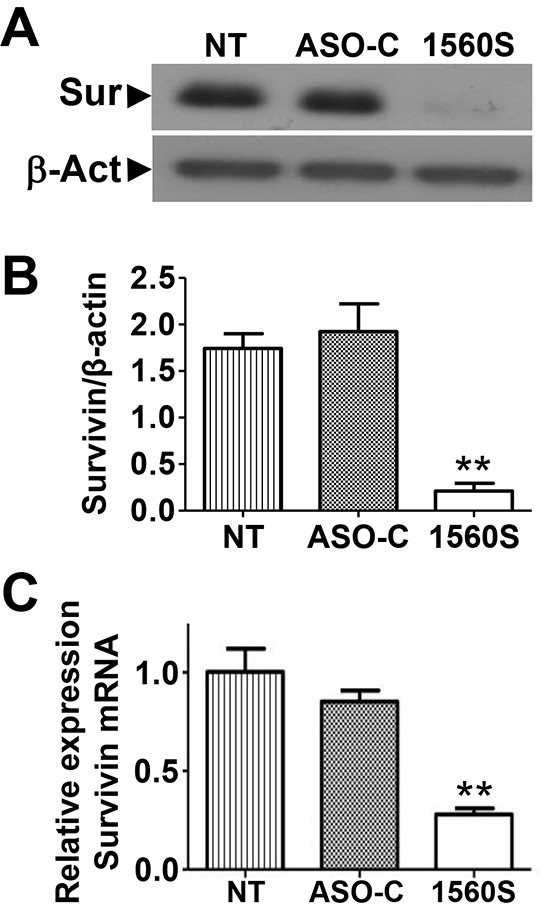
ASK induces downregulation of survivin **A.** B16F10 cells were seeded in 6-well plates at 10^5^ cells/well and transfected the next day with 200 nM ASO-C or ASO-1560S or left untreated (NT). At 24 h, cells were harvested and analyzed by Western blot. Survivin was drastically decreased by ASK, compared to controls (NT and ASO-C). **B.** A triplicate densitometric analysis of the experiment in (A) indicated that ASK induced around 90% decrease in survivin compared to controls (**p<0.001). **C.** Quantitative RT-PCR indicated that survivin mRNA is reduced to around 30% by ASK, compared to controls (**p<0.01).

Apoptotic cell death depends on the counterbalance between pro- and anti-apoptotic factors [28, 29]. Survivin, a member of the inhibitor of apoptosis (IAP) family plays an important cytoprotective function in cancer cells and is upregulated in virtually all human cancers, including melanoma [11–15]. Therefore, we asked whether ASK in B16F10 cells would affect survivin levels. Cells were transfected with ASO-C or ASO 1560S or left untreated for 24 h, harvested, lysed and subjected to Western blot using β-actin as loading control. ASK induced a marked reduction in survivin, compared to controls (Figure 4A). Densitometric analysis of blots from three independent experiments indicated that levels of survivin were reduced by about 90% (Figure 4B). Survivin mRNA levels also decreased after treatment with ASO-1560S, but less markedly than the protein (Figure 4C).

### Inhibition of tumorigenicity, stemness and invasiveness capacity of melanoma cells

**Figure 5:**
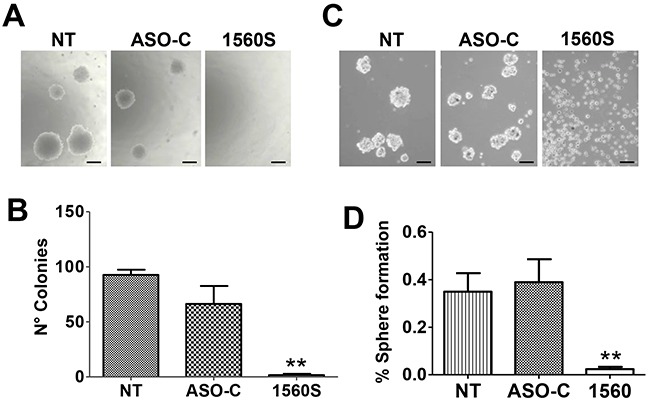
ASK reduces tumorigenic and invasive potential of B16F10 cells **A.** ASK inhibits anchorage-independent growth. B16F10 cells were transfected for 48h with 200 nM ASO-C or ASO-1560S or left untreated. After harvesting and counting, 2000 Tb^−^ cells were seeded in soft agar and colony formation was determined after 21 days in culture (see Materials and Methods). Bars = 100 μm. **B.** A triplicate assay indicated that ASK induced an inhibition of colony formation of over 90% compared to ASO-C (**p<0.005). Error bars represent average ± s.d. **C.** ASK induces inhibition of stemness. B16F10 cells were transfected as in (A) for 48 h and after harvesting, 4000 Tb^−^ cells were seeded on an agarose-coated plate and cultured for 10-11 days, when spheres >70 μm were scored. Bars = 100 μm. **D.** A triplicate analysis shows that upon transfection with ASO-1560S, the efficiency of sphere formation was less than 0.03%, compared to 0.35-0.4% in controls (**p<0.005).

A noteworthy hallmark of cancer cells is their anchorage-independent growth capacity, thus colony formation in soft agar is considered a parameter of tumorigenicity [30]. To determine whether ASK affects this property, B16F10 cells were transfected with ASO-1560S or ASO-C or left untreated (NT), for 48 h. Cells were then harvested, counted and 2000 Tb-negative cells per well were seeded into 12-well plates on soft agar, in triplicate. Colonies >100 μm in diameter were scored at 3 weeks in culture. ASK induced a drastic inhibition of colony formation (Figure 5A, 5B). Next, we determined the effect of ASK on stemness of B16F10 cells by sphere formation capacity [31]. After a 48 h treatment as described above, cells attached to the plate were harvested, counted and 4000 Tb-negative cells per well were seeded into 12-well agarose-coated plates. After 10-12 days in culture, spheres >70 μm in diameter were scored. Untreated or ASO-C-transfected cells formed spheres with 0.35 - 0.4% efficiency. Remarkably, sphere formation efficiency of cells transfected with ASO-1560S was less than 0.03% (Figure 5C, 5D).

**Figure 6:**
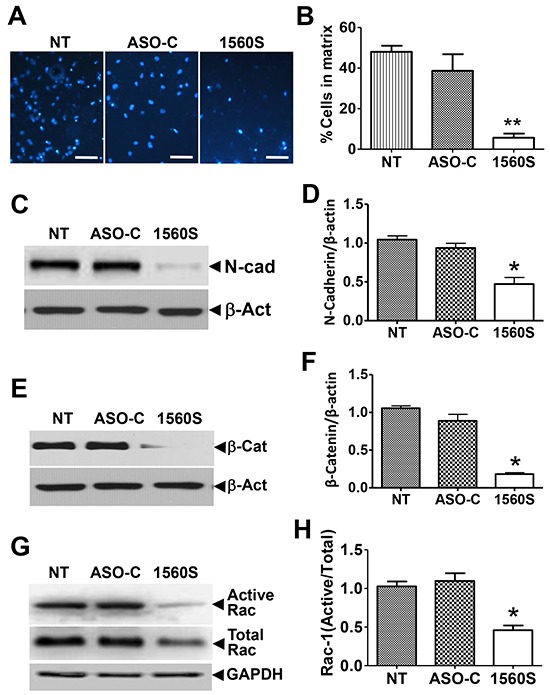
ASK reduces invasiveness parameters **A.** For matrigel invasion assay, 10^5^ B16F10 cells were transfected with ASO-1560S or ASO-C or left untreated for 48 h. Cells were collected and seeded onto the upper insert. After 24 h, the insert was stained with DAPI and invading cells were scored. **B.** A triplicate analysis of the experiment in (A) showed that ASK induces a drastic inhibition of B16F10 invasion (**p<0.005). **C.** B16F10 cells treated as in (A) for 48 h were collected and total lysate was subjected to Western blot with N-cadherin antibody. Representative blot shows a strong decrease in N-cadherin by ASK. **D.** A triplicate analysis shows a 50-60% downregulation of N-cadherin, compared to controls (**p<0.005). Error bars represent average ± s.d. **E.** Western blot of β-catenin shows a marked reduction in this protein in 1560S-treated cells. **F.** A triplicate analysis shows about an 80% reduction of β-catenin, compared to controls (*p<0.002). **G.** Cells treated as in (A) were collected and total supernatant was subjected to pull-down assay for active Rac-1 and precipitates were analyzed by Western blot. The portion of active Rac-1 was markedly reduced by ASO-1560S. **H.** A triplicate analysis showed that the proportion of active Rac-1 was reduced to about half of controls, in cells treated with 1560S (**p<0.001).

Furthermore, we found that the capacity of B16F10 cell invasion was inhibited around 8-10 times by ASK as compared to controls, in a Matrigel invasion assay (Figure 6A, 6B). ASK also induced a significant reduction in the levels of the epithelial-mesenchymal transition marker, N-cadherin [32] (Figure 6C, 6D), suggesting that the treatment negatively affects metastatic capacity. This notion is further supported by the marked reduction in levels of β-catenin [33] (Figure 6E, 6F) and the active form of Rac-1 [34] (Figure 6G, 6H).

### ASK *in vivo* induces inhibition of melanoma tumor growth and metastasis

**Figure 7:**
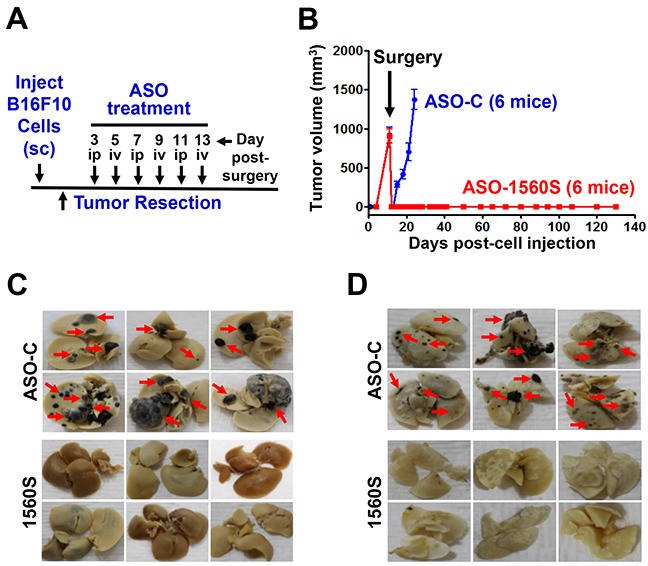
ASK *in vivo* inhibits tumor growth and metastasis **A.** Scheme of the syngeneic assay. **B.** Twelve C57BL/6 mice were injected subcutaneously with 100,000 B16F10 cells and at 11-12 days post-cell injection mice exhibited tumors between 700 and 1,000 mm^3^. The mice were randomized into two groups of six mice with similar tumor volume and, after anesthesia, tumors were surgically removed. Starting day 3 post-surgery, mice received 6 injections of 250 μl of saline containing 100 μg of ASO-C or ASO-1560S, alternating between ip and iv (A). At day 12 post-surgery, mice receiving ASO-C showed tumor relapse and were sacrificed under anesthesia. Tumors, lungs and livers were excised and fixed in formalin. No relapse was observed in the mice receiving ASO-1560S up to day 120 post-surgery, when they were euthanized under anesthesia and lungs and livers were collected and fixed in formalin. The livers **C.** and lungs **D.** of mice treated with ASO-C exhibited multiple metastasis nodules of different sizes (red arrows). In contrast, at 120 days post-surgery, livers (C) and lungs (D) were free of metastasis in mice treated with ASO-1560S.

Afterwards, we applied an experimental approach consisting of surgical removal of melanoma tumors followed by ASK treatment, similar to the protocol used in the clinical guidelines of human melanoma therapy (Figure 7A) [15–19]. B16F10 cells (10^5^ cells/mouse) were injected sc on the right flank of C57BL/6 mice. About 11-12 days post-injection, mice developed tumors between 700-1000 mm^3^ (Figure 7B). Mice were randomly divided into two groups with similar tumor volumes. Tumors were surgically resected under anesthesia and, starting 3 days post-surgery, mice received on alternating days 3 iv and 3 ip injections of 250 μl saline containing 100 μg of ASO-1560S or ASO-C (Figure 7A, arrows). ASO injections were carried out in blind and tumor growth was measured three times a week with a caliper. A representative experiment shows that primary tumor relapse was observed about 3 days post-surgery in mice treated with ASO-C and tumor volumes around 1,500 mm^3^ were reached on day 12 post-surgery (Figure 7B). These mice were euthanized and tumors and organs were collected and fixed. Remarkably however, no relapse was observed in mice treated with ASO-1560S targeted to the mASncmtRNAs. At 120 days post-surgery, mice appeared healthy, without palpable tumors (Figure 7B) and were euthanized to collect organs. In contrast to control mice, which displayed numerous metastatic nodules in livers and lungs (Figure 7C, 7D, red arrows), organs from mice treated with ASO-1560S appeared normal and devoid of visible metastatic nodules (Figure 7C, 7D).

**Figure 8:**
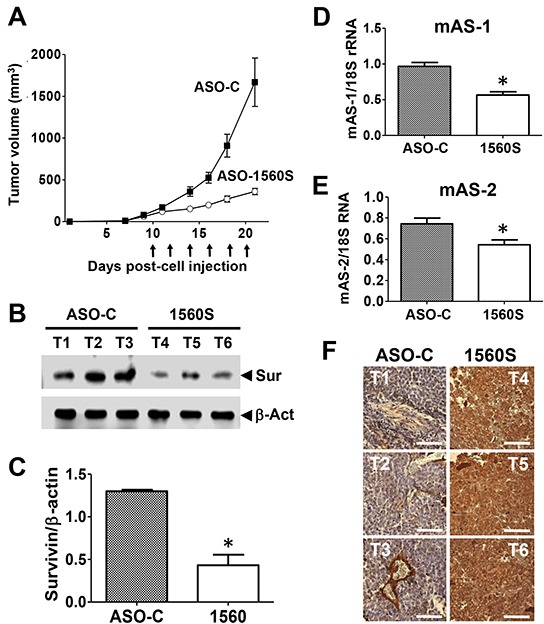
ASK *in vivo* retards tumor growth through antisense effect **A.** ASO-1560S induces tumor growth inhibition. Ten mice were injected subcutaneously with 100,000 B16F10 cells. On day 10 post-cell injection, tumor volumes reached about 100 mm^3^, mice were randomized into two groups of 6 mice each and were ip injected every other day with 250 μl saline containing 100 μg of ASO-C or ASO-1560S, 6 injections in total. Tumor volumes were monitored with a caliper. The following day after the last injection, tumors were excised and processed to obtain total protein, RNA and tissue sections (see Materials and Methods). **B.** Representative blots showing that treatment with ASO-1560S inhibits survivin expression. T1-3 tumors from ASO-C-treated mice; T4-6, tumors from ASO-1560S-treated mice. Sur = survivin; β-act = β-actin, used as loading control. **C.** Densitometry of blots showed that survivin expression in ASO-1560S-treated tumors was about 30% of the control level (*p<0.002). **D, E.** Levels of mASncmtRNA-1 and −2 were determined by end-point RT-PCR using 18S rRNA as control. The ratio between mASncmtRNAs and 18S rRNA was determined by densitometry. Treatment with ASO-1560S induces a relative inhibition of the levels of mASncmtRNA-1 (**p<0.005) and mASncmtRNA-2 (*p<0.05) compared to ASO-C. Error bars represent average ± s.d. **F.** Chromogenic TUNEL assay of resected tumors shows augmented apoptosis in tumors from mice treated with ASO-1560S, compared to ASO-C. Bars = 100 μm.

Next, and to determine the antisense effect induced by ASK,we used a different approach. C57BL/6 mice were injected sc on the right flank with 10^5^ B16F10 cells and when tumors reached a volume of about 100 mm^3^ estimated with a caliper, mice were randomized into two groups, which received 3 intraperitoneal (ip) and 3 intravenous (iv) injections of 250 μl saline containing 100 μg of either ASO-C or ASO-1560S (Figure 8A). ASO-1560S treatment induced a marked inhibition of tumor growth compared with ASO-C-treated mice (Figure 8A). One day after the last treatment, mice were sacrificed under anesthesia and tumors were removed and stored in liquid N_2_ or fixed. Treatment with ASO-1560S induced downregulation of survivin (Figure 8B, 8C) and knockdown of the ASncmtRNA-1 and −2 (Figure 8D, 8E). In addition, TUNEL assay revealed a marked signal corresponding to DNA fragmentation in tumor sections from mice treated with ASO-1560S (brown color) as compared with control tumors (Figure 8F).

**Figure 9:**
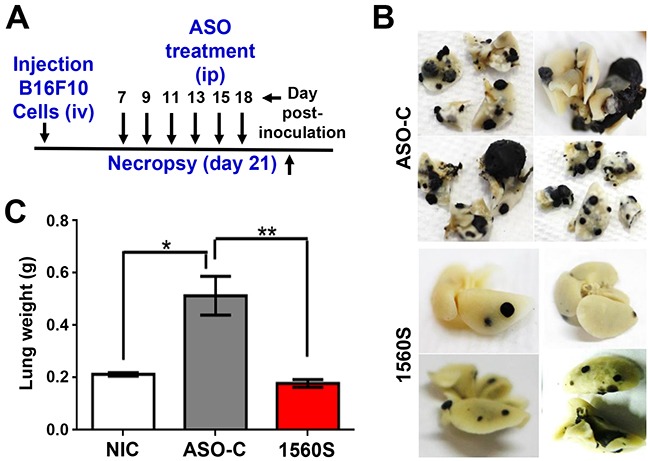
ASK *in vivo* decreases lung metastasis of B16F10 melanoma induced by tail vein injection of cells **A.** Scheme of the assay. Eight C57BL/6 mice were inoculated through the tail vein with 100,000 B16F10 cells. Mice were randomized into two groups of 4 mice each, which received 6 ip injections of 250 μl saline containing 100 μg of ASO-C or ASO-1560S on days 7, 9, 11, 13, 15 and 18 post-cell injection. On day 21 mice were sacrificed and lungs were collected and fixed in formalin. **B.** Lungs from mice treated with ASO-C exhibited multiple metastasis nodules of different sizes. In contrast, the lungs from ASO-1560S-treated mice treated displayed fewer and smaller nodules. **C.** Total weight of lungs from ASO-1560S-treated mice was comparable to control mice that had not been inoculated with B16F10 cells (non-inoculated controls; NIC), while the lungs of ASO-C-treated mice displayed an average weight increase of around 2.3 times.

In order to directly assess the effect of ASO-1560S on metastasis, we performed a “classic” *in vivo* metastasis assay by iv cell injection [35]. B16F10 cells (10^5^/animal) were injected through the tail vein of 8 C57BL/6 mice, in a total volume of 250 μl sterile saline. Afterwards, mice received 6 ip injections of 250 μl of sterile saline containing 100 μg ASO-C or ASO-1560S, on days 7, 9, 11, 13, 15 and 18 post-cell injection (Figure 9A). On day 21, mice were euthanized and lungs were collected and fixed (Figure 9A). As shown in Figure 9B, lungs of ASO-1560S-treated mice displayed a considerably lower number and size of melanoma nodules, compared to mice treated with ASO-C. The average weight of the lungs of the ASO-1560S group was comparable to that of lungs from control animals that had not been inoculated with B16F10 cells (non-inoculated controls, NIC), and less than half of ASO-C treated mice (Figure 9C).

Previous studies reported that ASOs with full phosphorothioate backbone might trigger inflammatory responses [36, 37]. To address this issue, three groups of 5 C57BL/6 mice were injected ip six times with 250 μl of saline alone or containing 100 μg of ASO-C or ASO-1560S. The day after the last injection, blood was collected from each mouse and the serum level of pro-inflammatory cytokines was determined. The serum levels of IL-6, IL-10, IL-12, IFNγ, TNFα and MCP1 in the ASO-1560S group were similar to controls (Supplementary Figure S4A). In addition, treatment with ASO-1560S did not affect body weight (Supplementary Figure S4B).

## DISCUSSION

Similar to the human transcripts, mSncmtRNA and the mASncmtRNAs contain stem-loop structures [1–3]. As discussed before, these transcripts in human cells and mouse cells including B16F10 cells exit the mitochondria and are found in the cytoplasm and the nucleus, suggesting that their functional role lies outside the organelle [9]. Most remarkable however is the identical expression pattern of these transcripts in both species. The mASncmtRNAs, expressed in normal proliferating cells, are downregulated in mouse tumor cells. Hence, at least in these two mammalian species, downregulation of the ASncmtRNAs seems to be a general hallmark of cancer [2, 4, 6]. The mechanism underlying this downregulation is not clear, but in Human Papilloma Virus-infected cells, it appears to be mediated by the E2 viral oncogene [3], hence in non-viral induced cancers, other cellular oncogenes could be involved in this event.

As we reported before for human tumor cells [6], we show here that knockdown of the ASncmtRNAs induces B16F10 cell death by classical hallmarks of apoptosis [27] without affecting viability of normal cells. We believe that the lower copy number of ASncmtRNA in tumor cells renders them more vulnerable to this knockdown treatment, although this hypothesis warrants further research. An intriguing issue is why the ASncmtRNAs are not suppressed completely in cancer cells. Perhaps the low copy number of ASncmtRNAs may be crucial to override cell cycle control mechanisms and thus to fuel cancer progression, by switching to a pro-survival function. This switch from tumor suppressor to pro-survival factor or oncogene has been described before. Such is the case for a missense mutation of p53 (mp53) existing in approximately 50% of human cancers, which confers oncogenic properties to p53 that contributes to cancer progression and metastasis. Moreover, silencing mp53 with RNAi induces massive apoptosis and diminishes the metastatic potential of cancer cells [38–43]. However, it is important to mention that the ASncmtRNAs are not mutated in tumor cells and exhibit the same sequence of these transcripts as in normal cells.

A pertinent question is what will happen in tumor cells harboring vectors encoding the ASncmtRNA-1 and −2. Would the ectopic expression of these transcripts render tumor cells resistant to ASK or become senescent (see below)? However, this experiment, albeit reasonable, is difficult to carry out. At present, all our efforts to clone these transcripts have been unsuccessful. We believe that this failure is due to replication slippage. DNA polymerases from different sources, including thermophilic enzymes, display a poor strand separation activity, resulting in replication slippage of the cDNA template [44–46].

As in human cancer cells, apoptosis of B16F10 cells induced by ASK is potentiated by downregulation of survivin, a cytoprotective factor of the IAP family, overexpressed in most cancers [11–15]. *In vitro* studies revealed that ASK of the human melanoma cell line SK-MEL-2 also induces downregulation of survivin [6]. However, the relative expression of survivin mRNA was not significantly affected by ASK [6]. Moreover, a luciferase reporter assay provides arguments to suggest that downregulation of survivin protein in SK-MEL-2 melanoma cells could be mediated by microRNAs (miRs), which have become increasingly important amongst epigenetic factors controlling tumorogenesis [47–49], probably generated by processing of the stem of the ASncmtRNAs by Dicer as a consequence of ASK [6]. Recently, Bianchessi et al. established a close relationship between replicative senescence of human endothelial cells and over-expression of the ASncmtRNA-2 [50]. Moreover, they reported that hsa-miR-1973 and hsa-miR-4485 are upregulated in senescent endothelial cells and propose that these miRs are derived from the ASncmtRNA-2 stem by Dicer processing [50]. Therefore, survivin, N-cadherin and and β-catenin downregulation by ASK in murine B16F10 cells may be the result of inhibition of translation of their mRNA by miRs generated from the stem of the mouse ASncmtRNAs. Although murine homologues of hsa-miR-1973 and hsa-miR-4485 cannot be currently found in miR databases, the corresponding regions on the mASncmtRNAs display sequence identities of 100% to human for miR-1973, 93% for miR-4485-5p and 90% for miR-4485-3p, suggesting that these segments on the double-stranded region of the ASncmtRNAs could give rise to murine homologues of the corresponding miRs. Indeed, we found that following ASK, MDA-MB-231 (human breast carcinoma) cells with ASO-1537S [6] for 24 h, hsa-miR-1973 and hsa-miR-4485 are overexpressed about 7-10 times compared to control cells (M. Briones and C. Fitzpatrick, unpublished results). Moreover, ASK triggers upregulation of approximately 12 previously described nuclear-encoded miRs plus 3 miRs hypothetically derived from the ASncmtRNAs. Transfection of hsa-miR-4485-3p results in downregulation of survivin and N-cadherin levels in MDA-MB-231 cells (Fitzpatrick and Briones, unpublished results).

Our *in vivo* studies in a syngeneic normal setting show that ASK inhibits B16F10 tumor growth and precludes tumor recurrence. In one representative *in vivo* assay, ASO-1560S induced a marked inhibition of tumor growth, confirmed knockdown of the ASncmtRNAs in resected tumors, inhibition of survivin expression and induction of TUNEL positive cells in tumors (Figure 8). We assayed a preclinical protocol involving surgical resection of subcutaneous melanoma tumors followed by systemic administration of ASO-1560S to induce knockdown of the mASncmtRNAs. Surgery is the only curative treatment for melanoma [51] and the preclinical protocol used in this work is similar to clinical practice guidelines, which involve surgical resection of the melanoma lesion followed by systemic treatment [16–20, 51]. Remarkably, there was no visible sign of lung or liver metastasis at 120 days since the beginning of treatment with ASO-1560S, although one cannot discard the possibility of micro-metastasis (Figure 7). In a different approach, in which we generated metastasis directly by inoculation of melanoma cells through the tail vein, ASO-1560S significantly reduced the number of metastatic nodules in the lungs, as well as their size (Figure 9). The fact that we did observe nodules in the therapeutic group, in contrast with the surgery assay, could be explained by the possibility of a higher number of circulating tumor cells in this approach, as opposed to cells originated from the primary tumor. Metastasis is a complex process that requires several factors (proteins and microRNAs) that cooperatively induce the invasive capacity of tumor cells [52, 53]. Among these factors, survivin, besides its participation as anti-apoptotic factor and in cell division, is an active player in metastasis [54, 55]. On the other hand, epithelial-mesenchymal transition (EMT) requires a switch from E-cadherin to N-cadherin [56, 57]. Indeed, here we show that ASK induces inhibition of the invasive capacity of B16F10 cells (Figure 6A, 6B), together with downregulation of survivin (Figure 4A, 4B), N-cadherin (Figure 6C, 6D) and β-catenin (Figure 6E, 6F) and a decrease in the activity of Rac-1 (Figure 6G, 6H), which may explain the decrease in metastatic potential *in vivo*.

In addition, ASO-1560S treatment neither elicited inflammatory response nor affected body weight (Supplementary Figure S4). Together with our previous reports [1–3, 6], the present results are a new contribution to the understanding of the relationship between melanoma and the ASncmtRNAs, new members of the large family of lncRNAs [58–60]. To our knowledge, the ASncmtRNAs are potentially the first non-nuclear-encoded target for treatment of melanoma.

Treatments were performed with 100 μg ASO-1560S (5 mg/kg body weight) per dose and without the use of any special carrier. An important question is how ASO-1560S, without any type of carrier is taken-up *in vivo* by the melanoma cells. There is good evidence that naked ASO is taken-up *in vivo* without the need of carriers or liposomes. Koller et al. identified the adaptor protein AP2M1 as being central to the uptake of ASO by an endocytotic process, independent of clathrin and caveolin, in mouse liver [61]. Mipomersen is an ASO that inhibits apoliprotein B synthesis in patients with severe hypercholesterolemia. This drug, approved by the FDA, is injected once a week as an aqueous solution [62, 63]. Therefore, it is reasonable to expect that the adaptor protein AP2M1 mediate the *in vivo* uptake of ASO-1560S in B16F10 melanoma cells. Indeed, as discussed before, ASO-1560S induces *in vivo* downregulation of ASncmtRNAs and survivin expression, in addition to DNA fragmentation (TUNEL assay) (Figure 8B-8F). Therefore, these results can only be explained by the incorporation of ASO-1560 into the tumor cells.

Worldwide, the incidence of melanoma, the most deadly form of skin cancer, is increasing. In the United States, melanoma incidence has been increasing approximately 2.8% per year since 1981 [64, 65]. Prognosis for patients with metastatic melanoma remains poor and efforts are currently focused on new targets and immunotherapy [66–68]. Collectively, our preclinical results establish proof-of-concept that the ASncmtRNAs may constitute a potent target to develop a treatment for melanoma. Also, supporting the use of the ASncmtRNAs as targets for cancer therapy, we have obtained similarly promising results, concerning tumor growth and metastasis, in a syngeneic model of murine renal adenocarcinoma (RenCa cells in Balb/c mice; V. Borgna, unpublished results), suggesting the potentiality of this approach for treatment of different types of cancer. Finally, it is important to mention that the Food and Drug Administration (FDA) recently approved ASO-1537S, homologous to ASO-1560S, targeted to the loop of the human transcripts [6] and Phase I clinical trial is underway in the USA.

## MATERIALS AND METHODS

### Cell culture

Mouse embryonic fibroblasts (MEF; ATCC SCRC-1008), murine myoblasts (C2C12; ATCC CRL-1772) and murine renal adenocarcinoma (RenCa; ATCC CRL-2947) cells were purchased from ATCC and cultured according to ATCC guidelines. Both normal cell types, MEF and C2C12, were not cultured beyond passage 6. Murine melanoma cell lines B16F0 and B16F10 were a kind gift from Dr. Ian Hart (Queen Mary University of London), which were cultured in RPMI 1640 (Life Technologies), supplemented with 10% fetal calf serum (Hyclone), 2 mM glutamine, 50 U/ml penicillin and 50 μg/ml streptomycin. Upon arrival, RenCa, B16F0 and B16F10 cells were expanded and frozen in liquid nitrogen at low passage number. After resuscitation, cells were not passaged beyond 6 months. The pigmented B16F0 and F10 lines were analyzed by determination of melanin content [69, 70] and tyrosinase activity [70–72], in order to confirm their melanocytic lineage. Differentiation of C2C12 cells into myotubules was induced for 4 days on 80% confluent cultures, by replacing normal growth media for DMEM supplemented with 2 mM glutamine, 50 U/ml penicillin, 50 μg/ml streptomycin, 0.1 mM NEAA and 5% horse serum (Life Technologies). All cultures were maintained at 37°C under a 5% CO_2_ atmosphere. Cultures were checked periodically for mycoplasma using the EZ-PCR Mycoplasma Test Kit (Biological Industries).

### In situ hybridization

Chromogenic *in situ* hybridization (ISH) of murine cells and tissues for detection of mSncmtRNA and mASncmtRNAs was performed as described before [1–3, 6], using probes 11S (5′ACCGTGCAAAGGTAGCATAATCA) and 11AS (5′TGATTATGCTACCTTTGCACGGT) (Figure1A).

### Cell transfection

Antisense oligonucleotides (ASOs) used in this study were synthesized by IDT or BioSearch Inc. with 100% phosphorothioate (PS) linkages [22]. The ASOs utilized were ASO-1560S (5′CACCCTCTAA CCTAGAGAAG) and control ASO or ASO-C (5′AGGTG GAGTGGATTGGGG). Transfection efficiency was confirmed with the same ASOs, labeled at the 5′ end with Alexa fluor 488. ASOs were dissolved in a culture hood in sterile physiological saline and filtered though a 0.2 μm non-pyrogenic filter (Pall Life Science Acrodisc, NY) into sterile tubes. Concentration was measured spectrophotometrically at 260 nm and stock solutions were stored at −80°C. Appropriate dilutions were prepared freshly in sterile saline. For ASO treatments, cells were seeded into 12-well plates (Nunc) at 25,000-50,000 cells/well and transfected the next day with 50-200 nM ASO, using Lipofectamine 2000 (Invitrogen), according to manufacturer's directions. Transfection was allowed to proceed for 24-48 h under normal culture conditions.

### Proliferation and cell viability

Cell number and viability was determined by Trypan blue (Tb) or propidium iodide (PI) exclusion, as described before [6].

### Conventional and quantitative RT-PCR amplification

RNA extraction, RNase digestion, cDNA synthesis and conventional PCR were carried out essentially as described [1–3, 6]. The murine ncmtRNAs were characterized by PCR-walking as described for human cells [1, 2]. For mSncmtRNA, reverse primer 1 (5′TTATATTTGTGTAGGGCTAGGGCT) was used in combination with forward primers 2 (5′GGGTTTCTAGCTGTAATTCTTT), 3(5′CTATTTTGCC ACATAGACGAGT), 4 (5′GAGCTGTCCCTCTTTTGGC TAT), 5 (5′TTGGTGGCTGCTTTTAGGCCTA), 6 (5′ACT CATACTAACAGTGTTGCAT), 7 (5′AGGTGGATTATTT ATAGTGTGA), 8 (5′TTTTTGGTAAACAGGCGGGGTT) and 9 (5′TGATTATGCTACCTTTGCACGGT). Primers used for amplification of the mASncmtRNAs were reverse primer 12 (5′TATATACGTACACCCTCTAACCTA) and forward primers 13 (5′CAATCCAGGTCGGTTTCTATCT), 14 (5′TC CTCCGAATGATTATAACCTA), 15 (5′TCCTCCGAATG ATTATAACCTA), 16 (5′CCTAATGGCCCAAAAACT ATA), 17 (5′AATAAGACGAGAAGACCCTATGGAG), 18 (5′AGTGAAGAGGCTGAAATATAAT), 19 (5′ACCGT GCAAAGGTAGCATAATCA), 20 (5′AACTTCTCTGTTAA CCCAACAC), 21 (5′TAGGCCTAAAAGCAGCCACCAA) and 22 (5′TTATAGCCAAAAGAGGGACAGC). Double- stranded regions were amplified using primers 10 (5′CTA AAAGAATTACAGCTAGAAACC) and 4 for mSncmtRNA and primers 23 (5′TGATTATGCTACCTTTGCACGGT) and 21 for mASncmtRNA. PCR amplification consisted of 5 min at 95°C, followed by 35 cycles of 95°C for 1 min, 55°C for 1 min and 72°C for 45 s. Amplification of GAPDH mRNA was used as a control for end-point PCR (forward primer 5′GATGCCCCCATGTTTGTGAT; reverse primer 5′GGTCATGAGCCCTTCCACAAT). The 18S rRNA (forward primer 5′GTAACCCGTTGAACCCCATT; reverse primer 5′CATCCAATCGGTAGTAGCG), also used as control, was amplified for only 15 cycles. Quantitative RT-PCR of survivin mRNA was performed essentially as described before [6], with forward primer 5′GAGGC TGGCTTCATCCACTGCCC and reverse primer 5′GACAA TGCTTGCCACTCAGGTGGG. The results were normalized against the murine mRNAs of HPRT (forward primer 5′GTCCCAGCGTCGTGATTAGC; reverse primer 5′TCAT GACATCTCGAGCAAGTCTTT) and RPS29 (forward primer 5′GCAAGATGGGTCACCAGCAG; reverse primer 5′GACATAGGCTTCATTAAGTTGGAC).

### Northern blot

Total RNA from MEFs or mouse testis (10 μg) was electrophoresed on a denaturing 1% agarose gel containing 2.2 M formaldehyde, at 80V for 3 h. After ethidium bromide staining, the gel was soaked for 10 min in 5x SSC/10 mM NaOH and transferred to a nylon membrane in the same buffer, for 3.5 h at RT. Membranes were washed briefly in gel-running buffer and cross-linked under UV light. Pre-hybridization, hybridization and streptavidin-AP detection was performed using the Detector AP Chemiluminescent Blotting Kit (KPL) according to manufacturers'directions. Briefly, membranes were incubated overnight at 42°C with 330 ng/ml biotinylated probe in hybridization buffer, washed twice for 15 min in 2X SSPE/0.5% SDS at 25°C and once for 5 min in 1x SSPE. Membranes were then blocked for 1 h and incubated 1 h in a 1:10,000 dilution of streptavidin-AP. After 3 washes for 5 min in washing buffer and 2 washes in assay buffer for 2 min each, membranes were then subjected to chemiluminiscent detection with CDP star (KPL). The probes utilized were complementary to the junction region between the 16S sequence and the IR of the mSncmtRNA (5′GTGTAGGGCTAGGGCTAGGATTAG-TGCCGCAAGGGAAAGATGAAAGACTA), mASncmt RNA-1 (5′CGTACACCCTCTAACCTAGAGAAGGTT GTACGTATATATTTTATTTAGATTTTATTC) or mASnc mtRNA-2 (5′ACGTACACCCTCTAACCTAGAGAAGGTTATTAGGTTTAATAAATTAAATAGATAAGT) labeled with biotin at the 5′ and 3′ ends (IDT).

### Mitochondrial membrane depolarization

Depolarization of the mitochondrial membrane was determined as described before [6].

### Preparation of whole cell extracts and subcellular fractionation

Whole cell extracts and cytosolic fraction were obtained as described [6]. Protein concentration was quantified with the Bradford microplate-system Gen5TM EPOCH (BioTeK) [6].

### Western blot

Proteins (30 μg/lane) were resolved by SDS-PAGE and transferred to polyvinylidene difluoride (PVDF) membranes. Membranes were probed with antibodies against cytochrome c (rabbit polyclonal; Cell Signaling; 1:1000), survivin (rabbit polyclonal; R&D systems; 1:1000), N-cadherin (Santa Cruz Biotechnology; 1:1000), β-catenin (Cell Signaling; 1:1000) or Rac-1 (Cell Signaling; 1:3000) and revealed with peroxidase-labeled anti-mouse or anti-rabbit IgG (Calbiochem; 1:5000). Blots were detected with the EZ-ECL system (Biological Industries). Mouse monoclonal anti-β-actin (Sigma-Aldrich; 1:4000) was used as loading control. The intensity of each band was quantified using ImageJ software (NIH).

### Rac-1 activity assay

Determination of the fraction of active Rac was carried out by pull-down assay, as described [73]. Results were expressed as Active/Total Rac.

### DNA fragmentation

DNA fragmentation was evaluated by Dead-End^TM^ Fluorometric TUNEL kit (Promega) [6], and by Flow cytometry quantification of hypodiploid cells (sub-G1 fraction), as described [6].

### Determination of phosphatidylserine exposure

Phosphatidylserine (PS) exposure was determined by Annexin-V binding using the APOtarget kit (Invitrogen), according to manufacturer's directions, followed by Flow cytometry [6].

### Caspase activation

Caspase activation was determined using the fluorogenic caspase inhibitor CaspACE™ FITC-VAD-fmk (Promega), as described before [6].

### Colony, sphere formation and invasion assay

Anchorage-independent cell growth was determined by colony formation in soft agar as described [6, 30]. Untreated B16F10 cells (NT) or cells transfected with 150 nM ASO-C or ASO-1560S for 48 h were harvested and 200 Tb-negative cells were seeded into 12 well-plates in soft agar. Formation of colonies >100 μm in diameter was scored at 2-3 weeks. To determine stemness [31], 1000 Tb-negative B16F10 cells treated as described, for 24 h, were seeded into 2% agarose-coated 12-well-plates. Spheres >70 μm in diameter were scored at 10-12 days. For matrigel invasion assay,10^5^cells treated as above for 48 h were seeded over Matrigel-coated inserts (Matrigel Invasion Chamber 8.0 lm; BD Biosciences). After 24 h, inserts were fixed in cold methanol, membranes were mounted in Mowiol, and observed under a light microscope at 40x [73]. At least 10 fields were evaluated.

### TUNEL assay

Colorimetric TUNEL assay to determine apoptosis in tumor tissues was carried out on B16F10 tumor sections obtained as described below. The TUNEL procedure was performed using DeadEnd™ colorimetric apoptosis detection system (Promega), according to manufacturer's instructions. As positive control, DNAse I-treated sections were included.

### Animal studies

Animal experiments were conducted in accordance with the guidelines of Comisión Nacional de Investigación Científica y Tecnológica (Conicyt), Chile and approved by the Ethical Committee of Fundación Ciencia & Vida. C57BL/6 mice (The Jackson Laboratory) were maintained under specific pathogen-free conditions and used at 6–8 weeks of age. To determine the antitumor effect of ASO-1560S or ASO-C, 10 C57BL/6 mice were injected subcutaneously (sc) with 10^5^ B16F10 cells on the right flank. When tumors reached a volume about 100 mm^3^, mice were randomized into two groups of 5, which received 6 intraperitoneal (ip) injections of 100 μg ASO-C or ASO-1560S, every other day. Two days after the last injection, mice were euthanized under anesthesia and tumors were removed and flash-frozen in liquid N_2_. Tumors were processed to obtain tissue sections, total proteins and total RNA. The post-surgery metastasis study was conducted by generation of subcutaneous tumors, followed by surgical removal of tumors and ASO injection. For this purpose, 10^5^ B16F10 cells in 250 μl saline were injected subcutaneously on the right flank of 12 C57BL/6 mice. Tumor growth was monitored every other day with a caliper and tumor volumes were calculated on the basis of the formula: tumor volume = L x W^2^ x 0.5236, where L is mid-axis length and W is mid-axis width. At 11-12 days post-cell injection, tumor volumes reached 700-1000 mm^3^ and mice were randomized into two groups of 6: the ASO-C group and the ASO-1560S group. Tumors were surgically resected under anesthesia and the wound was washed once with 250 μl saline containing 100 μg of ASO-1560S or ASO-C. After suture, a bolus of 250 μl saline containing 100 μg of ASO-C or ASO-1560S was applied into the cavity left by the tumor. Starting 3 days post-surgery, mice received on alternating days 3 intravenous (iv) and 3 ip injections of 100 μg ASO-1560S or ASO-C in 250 μl saline. All ASO applications were done in blind. After the last injection, tumor growth was measured twice a week with a caliper. At the end of the experiment, mice were sacrificed and lungs and liver were removed and fixed in 10% formalin. Metastatic melanoma nodules were scored under a dissecting microscope. For direct lung metastasis generation, 8 C57BL/6 mice were inoculated through the tail vein with 250 μl sterile saline containing 10^5^ B16F10 cells per mouse. Mice were randomized into 2 groups of 4. On days 7, 9, 11, 13, 15 and 18 post-cell injection, mice received ip injections of 250 μl sterile saline containing 100 μg ASO-C or ASO-1560S. On day 21, mice were euthanized and lungs were collected and fixed in 10% formalin. Metastatic melanoma nodules were scored under a dissecting microscope.

### Cytokine ELISA

Determination of serum levels of interleukin-6 (IL-6), interleukin-10 (IL-10), interleukin-12 (IL-12), interferon-γ (IFN-γ), tumor necrosis factor-α (TNF-α) and monocyte chemoattractant protein-1 (MCP1) of mice treated with ASO-1560S or ASO-C or left untreated was carried out by sandwich ELISA (BD Biosciences, San Diego, CA), according to manufacturer's directions.

### Statistical analysis

Experimental results were analyzed by the Student's *t*-test. Significance (*p-*value) was set at the nominal level of *p*<0.05 or less.

## SUPPLEMENTARY FIGURES AND VIDEOS








