# Supplementary material for: Targeting antisense mitochondrial ncRNAs inhibits murine melanoma tumor growth and metastasis through reduction in survival and invasion factors
Source: Oncotarget. 2016 Aug 6;7(36):58331–50. doi: 10.18632/oncotarget.11110 (PMC5295434; doi:10.18632/oncotarget.11110)
Supplement: Supplementary file 1 [file oncotarget-07-58331-s001.pdf]

Targeting antisense mitochondrial ncRNAs inhibits murine melanoma tumor growth and metastasis through reduction in survival and invasion factors

SUPPLEMENTARY FIGURES AND VIDEOS

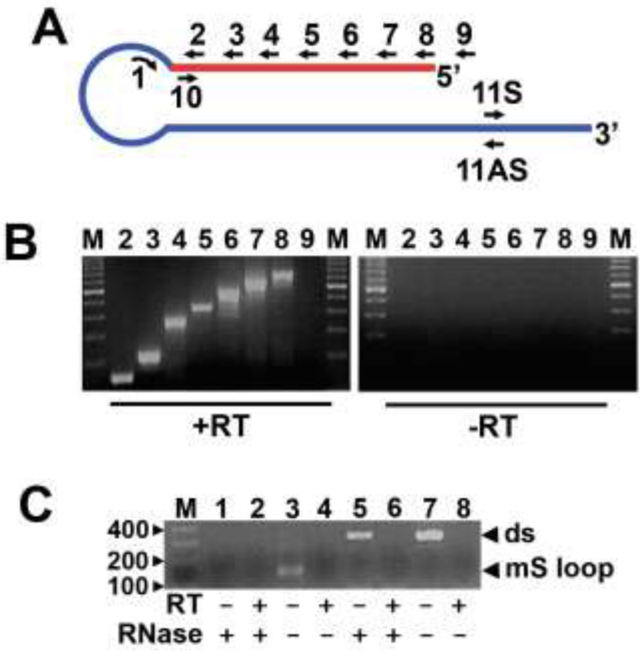

Supplementary Figure S1: Characterization of mouse SncmtRNA (mSncmtRNA). A. Amplification scheme of the mSncmtRNA representing the mouse mitochondrial 16S rRNA (blue line) linked at the 5' end to an IR (red line). The primers used for RT-PCR amplification (1 to 10) and probes (11S and 11AS) are indicated. **B.** Amplification of RNA from C2C12 cells by RT-PCR between primer 1 (reverse) and primers 2 to 8 (forward) generated a ladder of amplicons. No amplification was obtained without reverse transcriptase or with primer 9, indicating that the 5' of the transcripts is between primers 8 and 9. **C.** The double-stranded stem of the mSncmtRNA is resistant to RNase A digestion. Total RNA from C2C12 cells in 2x SSC was incubated without (-) or with (+) 10 µg/ml of RNase A for 5 min at 25°C. The RNA was recovered and amplified by RT-PCR using primers 1 and 2 (lanes 1-4) or primers 10 and 4 (lanes 5-8). An amplicon of 150 bp (lane 3), corresponding to the junction region between 16S rRNA and the IR (mS loop), was obtained only with untreated RNA. Amplification of a 350 bp fragment, corresponding to the double-stranded stem (ds) obtained with primers 10 and 4 was not affected by the nuclease treatment (lanes 5 and 7). M, 100-bp ladder.

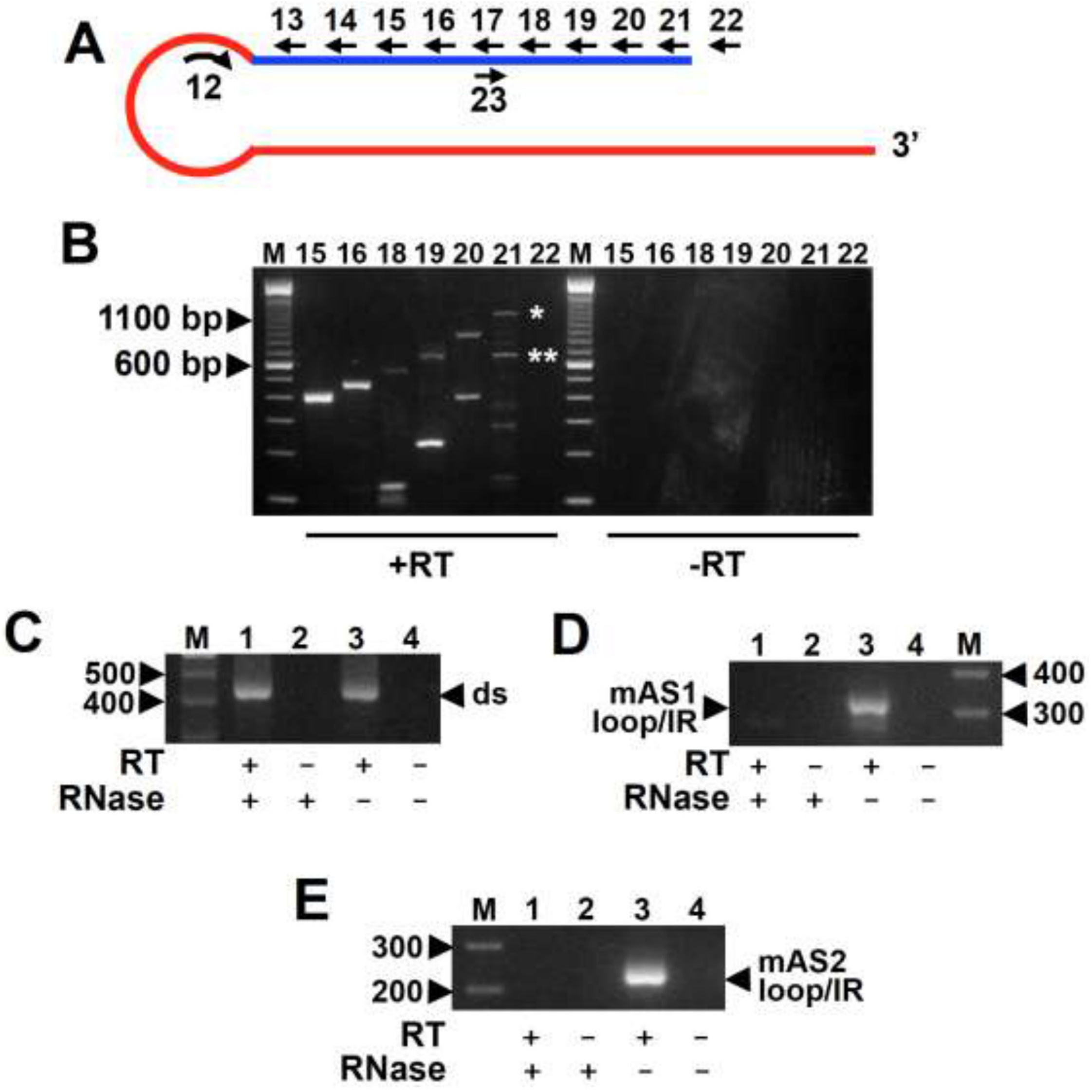

Supplementary Figure S2: Characterization of the mouse antisense ncmtRNAs (mASncmtRNAs). A. Amplification scheme of the mASncmtRNA representing the antisense mouse mitochondrial 16S RNA (red line) linked at the 5' to an IR (blue line). Primers used for RT-PCR amplification (12 to 23) are indicated. **B.** Amplicons were obtained by RT-PCR amplification of C2C12 cDNA using primer 12 (rev) in combination with forward primers 15, 16, 18, 19, 20 and 21. No amplification was obtained with primer 22 or without RT (-RT). M, 100-bp ladder. *amplicon corresponding to mASncmtRNA-1; **amplicon corresponding to mASncmtRNA-2. **C, D, E.** C2C12 RNA treated as in Supplementary Figure S1C was subjected to RT-PCR using primers 21 and 23 for the double-stranded region of both mASncmtRNAs **C.**, 12 and 14 for the loop/IR junction of mASncmtRNA-1 **D.** and 12 and 19 for the loop/IR junction of mASncmtRNA-2 **E.**. M, 100-bp ladder.

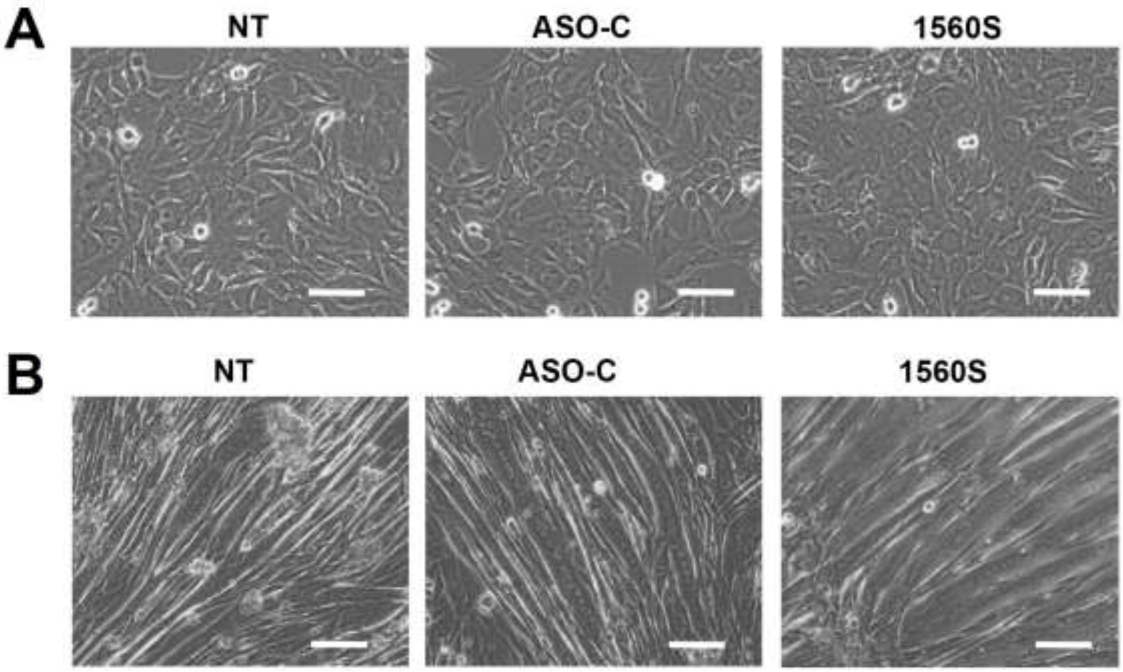

Supplementary Figure S3: ASK does not affect growth or morphology of C2C12 cells. A. C2C12 mouse myoblasts were treated as in Figure 2F for 3 days, displaying normal morphology and growth rate as controls. **B.** C2C12 cells differentiate normally into myotubules after ASK. C2C12 cells transfected for 3 days with ASO-1560S or ASO-C or untreated were washed and media was replaced with differentiation media (See Materials and Methods). Differentiation was allowed to proceed for 4 days. ASO-1560S-treated cells retained the ability to differentiate into morphologically normal myotubules, as for control cells. Bars = 50 μm.

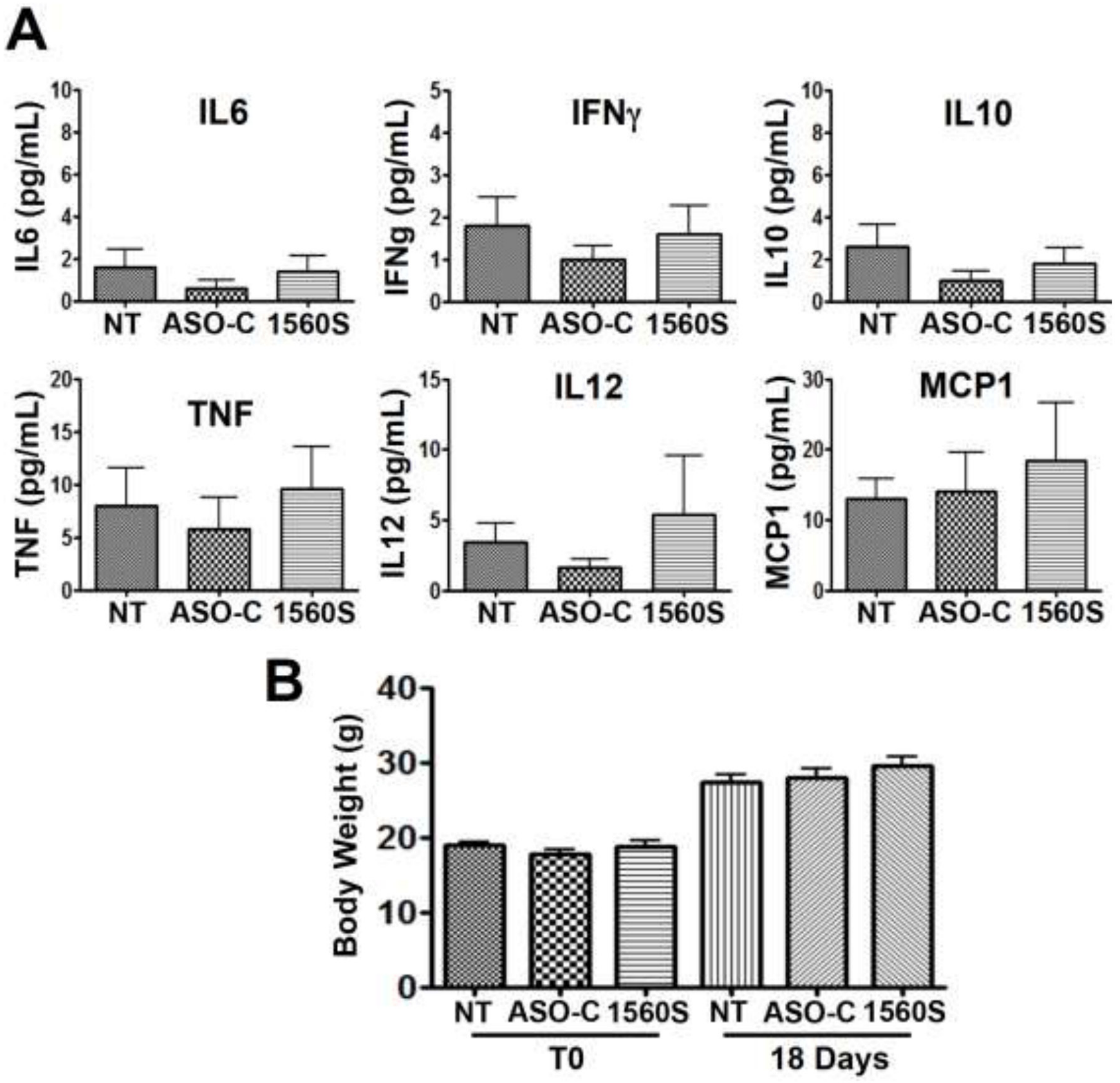

Supplementary Figure S4: ASK *in vivo* does not induce inflammatory response. A. Three groups of 5 C57BL/6 mice each were injected ip six times, every other day, with 250 µl of Saline (NT), or 250 µl of saline containing 100 µg of ASO-C or 100 µg of ASO-1537. After the last treatment, mice were weighed and blood was obtained from the tail vein. Cytokines IL6, IL10, IL12, INF-γ, TNF-β and MCP1 in serum were determined by ELISA. The cytokine levels in mice treated with ASO-1560S was similar to the controls NT and ASO-C. **B.** Body weight was not altered by treatment with ASO-1560S. Error bars represent average ± s.d.

SUPPLEMENTARY MATERIALS

Supplementary Videos: C2C12 cells were treated as described for Supplementary Figure 3b and 3c, and allowed to differentiate for 4 days in differentiation media (Materials and Methods), after which contraction of differentiated myotubules was recorded on an Olympus CKX-41 phase contrast microscope.

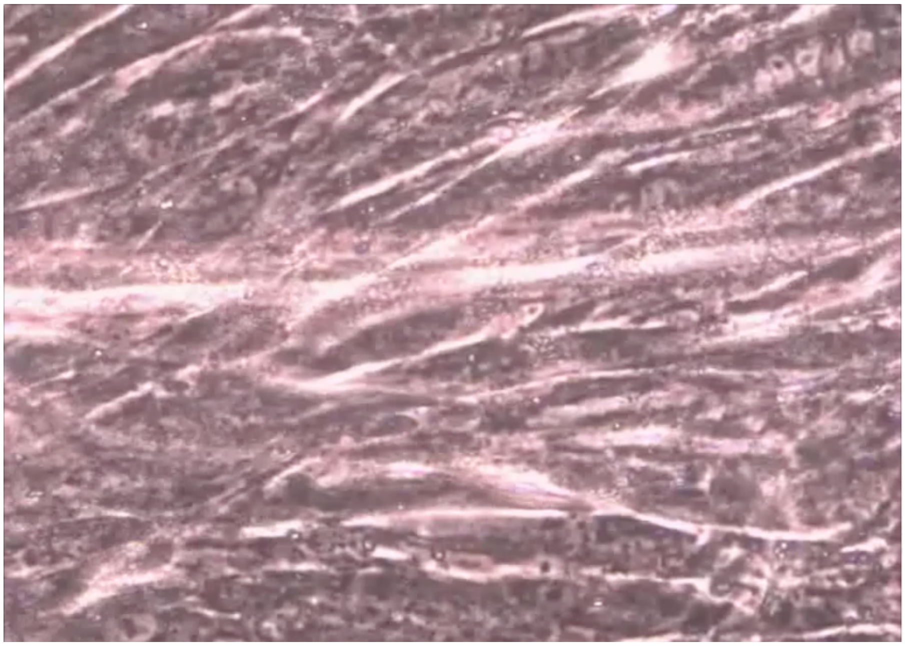

Supplementary Video S1: Myotubules differentiated from C2C12 cells treated with ASO-1560S.

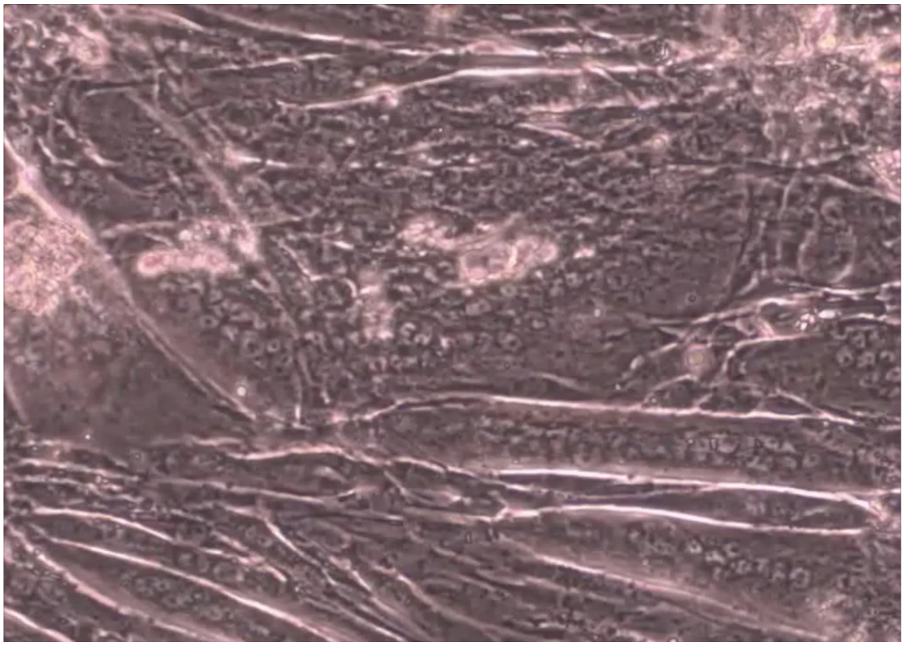

Supplementary Video S2: Myotubules differentiated from C2C12 cells treated with ASO-C.

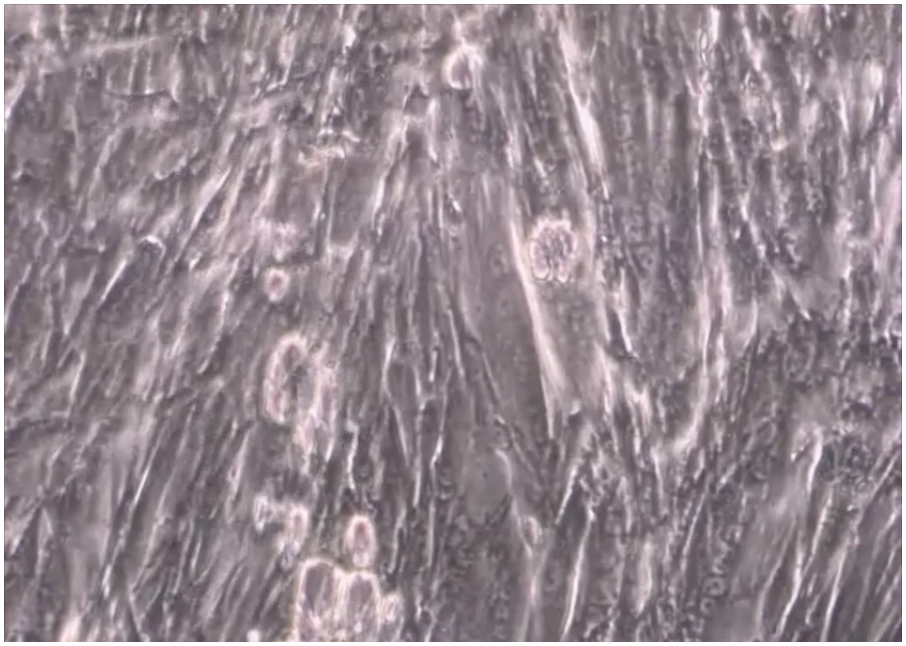

Supplementary Video S3: Myotubules differentiated from untreated C2C12 cells.
